# Supplementary material for: Functional dynamic prosthesis alignment maintained across varying footwear using a modular prosthetic ankle-feet system
Source: PLoS One. 2025 May 28;20(5):e0323647. doi: 10.1371/journal.pone.0323647 (PMC12118934; doi:10.1371/journal.pone.0323647)
Supplement: S2 Information File — (DOCX) [file pone.0323647.s002.docx]

Supporting Information File 1: additional participant demographics

| **ID** | **Age** | **Gender** | **Prosthesis Use (years)** | **Cause of Amputation** | **Height (m)** | **Mass (kg)** |
| --- | --- | --- | --- | --- | --- | --- |
| Participant 1 | 38 | Woman | 4 | Elective | 1.67 | 81.6 |
| Participant 2 | 47 | Woman | 36 | Trauma | 1.57 | 65.8 |
| Participant 3 | 29 | Woman | 5 | Trauma | 1.54 | 49.9 |
