# Supplementary material for: Functional dynamic prosthesis alignment maintained across varying footwear using a modular prosthetic ankle-feet system
Source: PLoS One. 2025 May 28;20(5):e0323647. doi: 10.1371/journal.pone.0323647 (PMC12118934; doi:10.1371/journal.pone.0323647)
Supplement: Attachment — Submitted filename: Response to Reviewers.pdf [file pone.0323647.s004.pdf]

VA | U.S. Department of Veterans Affairs
Veterans Health Administration
Minneapolis VA Health Care System

One Veterans Drive
Minneapolis, MN 55417
www.va.gov/minneapolis-health-care

Nicole R. Walker, MS, CPO
Rehabilitation & Engineering Center for Optimizing Veteran Engagement & Reintegration (RECOVER)
Minneapolis VA Health Care System
Nicole.Walker6@va.gov

2 December 2024

Dear Editors-in-Chief,

We greatly appreciate the reviewer's thoughtful evaluation of our manuscript. Please see our responses to the reviewer.

Reviewer 1:
"The authors specifically investigate the anterior posterior shift of the prosthesis user's rollover shape. There is no information stating why the anterior posterior shift is important and should be researched. The authors need to add a paragraph explaining why this is an important metric to investigate, including but not limited to what happens if the anterior posterior shift is too drastic."

Additional information and citations have been added to the introduction to further describe rollover shape and the anterior posterior shift (referred to as the x_0 location of the center of the arc). Additionally, further information has been included to describe the influence of heel height on the x_0 location, the primary variable of interest for this pilot work. See lines 48-58 and 65-75 in the tracked changes manuscript.

"One of the main points being made in the paper is that their system does not dramatically change rollover shape. However, it is not clear how sensitive roll over shape is to relevant variables in general (alignment, stiffness, etc.). For instance, is it possible that changes to prosthesis stiffness or alignment would similarly not impact roll-over shape, even though we know that they have other different effects on gait? Overall, the argument that rollover shape is little changed and thus the device works, is missing validation of the sensitivity of rollover shape to detect meaningful differences in gait / prosthesis mechanics. This should be addressed through an explicit comparison with other variables in prior published literature."

Citations reporting the sensitivity of rollover shape (and more specifically of the x_0 location) have been added in lines 55-58 in the tracked changes manuscript.

"The majority of the needed information is not directly in the paper but rather only found in the references which made reading this challenging. The RECOVER ankle-feet system is only briefly explained without enough information to know exactly how the system works. The authors need to add this information into this paper and not just rely on the reference. Furthermore, roll-over shape is not an outcome metric that is easy for most people to understand. Since rollover shape is the main outcome measure, the authors need to add sufficient detail in the paper about what it is and how it is found, not just relying on the references."

Additional detail describing the RECOVER ankle-feet system has been added in line 34-42 of the tracked changes manuscript. An additional figure (Figure 2) has been added to illustrate the ankle-foot rollover shape

as it applies to measuring this socket based metric, and additional information about rollover shape and arc center location have been added in lines 48-58 and 65-75 in the tracked changes manuscript.

"Prosthetic feet have changed measurably since 1986, therefore, it may not be correct to assume that the clinically acceptable parameter of 1.6 cm found in the Zahedi paper would hold true for prosthesis users today. The prosthesis users in the Zahedi paper only used Sach or uniaxial prosthetic feet from back then which cannot be fairly compared to newer flexible keel feet for those with the functional level of K2 or the energy storage and return (ESR) feet used by those with the functional level of K3. This may also hold true for the sockets, liners, and suspension that were used in the 1980's compared to today's technology. The authors may not be able to change this issue for this current publication but may be something to be aware of for their future research."

The authors certainly acknowledge that prosthetic feet technology has changed considerably since Zahedi's publication in 1986. However, this publication remains (to our knowledge) the most meaningful piece of literature exploring the clinically acceptable variability of anterior-posterior alignment among transtibial prosthesis users. An acknowledgment of the potential limitation of the age of Zahedi's publication has been added to the Discussion section in lines 217-223 in the tracked changes manuscript.

"The authors mention that there are "several products available that allow prosthesis users to adapt their alignment for different footwear" but then alignment becomes the focus, specifically how patients have a hard time aligning their own prosthesis correctly. Products and adjusting prosthesis alignment are different unless the product is a wrench. The authors should add a section explaining hydraulic feet, what these products are lacking, and how their system may be better for prosthetic users."

Additional information about currently available prosthetic feet technologies, including heel height adjustable feet and feet with hydraulic or microprocessor-controlled ankles, and their adaptability to heel height has been added to the discussion section in lines 187-189 and lines 194-198.

"For proper use of the RECOVER ankle-feet system, the prosthesis user would need a specific 3D printed foot for each of their shoes that have a unique heel height (in other words, multiple prosthetic feet). Some prosthesis users will be fine with this and some will not. The authors should consider mentioning this as a possible limitation."

An additional potential limitation to using the RECOVER ankle-feet system has been added in lines 210-217.

"The authors should add more references pertaining to current prosthetic feet used in clinic, alignment, biomechanics of prosthesis users, the impacts alignment can have on the users, and rollover shape."

We believe the additional information and citations provided in addressing the previous reviewer comments also include this request and offer greater clarity to the reader.

Sincerely,

Corresponding Author: Nicole Walker | Nicole.Walker6@va.gov | 612.467.3229
