# Supplementary material for: Functional dynamic prosthesis alignment maintained across varying footwear using a modular prosthetic ankle-feet system
Source: PLoS One. 2025 May 28;20(5):e0323647. doi: 10.1371/journal.pone.0323647 (PMC12118934; doi:10.1371/journal.pone.0323647)
Supplement: Attachment — Submitted filename: Response_to_Reviewers_auresp_2.pdf [file pone.0323647.s005.pdf]

VA | U.S. Department of Veterans Affairs
Veterans Health Administration
Minneapolis VA Health Care System

One Veterans Drive
Minneapolis, MN 55417
www.va.gov/minneapolis-health-care

Nicole R. Walker, MS, CPO
Rehabilitation & Engineering Center for Optimizing Veteran Engagement & Reintegration (RECOVER)
Minneapolis VA Health Care System
Nicole.Walker6@va.gov

8 April 2025

Dear Editors-in-Chief,

We greatly appreciate the reviewers' thoughtful evaluation of our manuscript. Please see our responses to the journal requirements and reviewer. No changes have been made to the manuscript content.

Journal Requirements:
Please review your reference list to ensure that it is complete and correct. If you have cited papers that have been retracted, please include the rationale for doing so in the manuscript text, or remove these references and replace them with relevant current references. Any changes to the reference list should be mentioned in the rebuttal letter that accompanies your revised manuscript. If you need to cite a retracted article, indicate the article's retracted status in the References list and also include a citation and full reference for the retraction notice.

All references have been reviewed and deemed up to date.

Reviewer 2:
"This revised manuscript offers more detail in areas lacking, specifically on RECOVER system and Figure 2."

Thank you for this feedback.

"I agree that evaluating frontal and transverse kinematics are warranted, as are moments and socket comfort across longer repeated durations."

We appreciate this feedback and will keep this in mind for future work.

"Roll over is an indices, however, moment of force may support your finding."

We appreciate this feedback and will expand our future work to explore ankle torque and torque-angle relationships as well as roll-over shapes.

"Is this system a component which could be ordered? Would it fall under any current L-Codes?"

Our group is working on technology transfer to an industry partner. The most appropriate reimbursement coding is also being determined.

Sincerely,

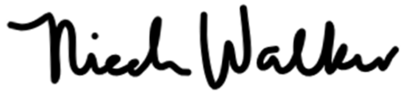

Corresponding Author: Nicole Walker | Nicole.Walker6@va.gov | 612.467.3229
